# Chemical and quality evaluation of Pacific white shrimp *Litopenaeus vannamei*: Influence of strains on flesh nutrition

**DOI:** 10.1002/fsn3.2457

**Published:** 2021-08-10

**Authors:** Xiao Li, Ying Wang, Hongyan Li, Xiaodong Jiang, Lei Ji, Tianhong Liu, Yuanqin Sun

**Affiliations:** ^1^ Marine Science Research Institute of Shandong Province Qingdao China; ^2^ Qingdao Quality Evaluation and Utilization Engineering Research Center for Aquatic Organism Qingdao China

**Keywords:** amino acid, fat acid, *Litopenaeus vannamei*, nutritional component, strains

## Abstract

The Pacific white shrimp, *Litopenaeus vannamei*, is an important fisheries resource in China. To investigate the differences in nutritional quality among strains, we analyzed and compared the basic muscle nutritional components, amino acid (hydrolyzed and free) compositions, and fatty acid compositions among four *L. vannamei* strains (Universal, KH‐1, Syaqua, and common). The result showed that under an efficiency aquaculture model, all four strains had high protein (21.1%‐22.3%) and low fat (0.8%‐1.1%). The Universal strain was highest in protein and fat as well as essential amino acid score (147.97). The Syaqua strain had the highest levels of polyunsaturated fatty acids (44.88%). The KH‐1 strain had the highest free amino acid content (2.68%), which contributes to the taste. Our findings revealed that strain‐specific variability exists in chemical composition of the shrimp *L. vannamei* under controlled condition, which may provide buying reference for consumers.

## 1 INTRODUCTION

The Pacific white shrimp, *Litopenaeus vannamei*, is the dominant farmed shrimp worldwide, representing one of the most common aquaculture species (Zhang et al., 2014). *L. vannamei* is an important commercial species in China, with a catch volume exceeding 114,370 tons in 2019 (FBMA, 2020). Pacific white shrimp is widely favored due to its superior flesh quality, delicious taste, and nutritional properties, and ease of cooking. Given the high market value, *L. vannamei* has emerged as one of the most valuable globally traded seafood products (Blythe et al., 2015).

*L. vannamei* is a euryhaline shrimp that is suitable for high‐density cultivation in seawater and salt‐fresh water. Suitable aquaculture species possess the characteristics of stress resistance, strong disease resistance, rapid growth, low cost, and high benefit (Li et al., 2016). China depends on imported shrimp, such as Syaqua from Thailand and Universal from Indonesia to obtain improved parent varieties (Li et al., 2016). However, some new strains have been bred in China, such as Kehai No.1 (KH‐1) and Zhongke No.1 (Chen et al., 2008; Huang et al., 2010). At present, research is mainly focused on germplasm selection (Hu et al., 2015; Luan et al., 2013). Few studies have documented the nutritional components of different *L. vannamei* strains.

Nutritional composition is an important index of germplasm evaluation for shrimp, providing a basis for resource development and genetic improvement (Ma et al., 2018). Different specifications, salinity conditions, aquaculture environment, and model have been reported to affect the nutritional composition and quality of *L. vannamei* (Li et al., 2020; Xu et al., 2015; Duan et al., 2017). However, these studies evaluated the same shrimp varieties under different conditions. Determining the nutritional components of different strains under the same cultivated condition can provide novel nutritional information to consumers.

The objective of this study is to evaluate and compare the basic muscle nutritional components, amino acid (hydrolyzed and free) compositions, and fatty acid compositions of four *L. vannamei* strains (Universal, KH‐1, Syaqua, and common) under the efficiency aquaculture model. Our findings will provide essential information for the comprehensive development, utilization, and promotion of *L. vannamei* strains.

## 2 MATERIALS AND METHODS

### 2.1 Experimental design

The 90‐day feeding trial was performed from May 2020 to August 2020. The experiment was conducted in a shrimp cultural farm (Binzhou, China). Prior to the experiment, juvenile shrimps (Universal, KH‐1, Syaqua, and common) with initial weights of 0.63 ± 0.02 g were temporarily reared for seven days to acclimate. Next, the shrimps were placed into 12 experimental ponds (30 m^3^) to constitute four groups in triplicate. The stocking density was about 300 individuals per square meter. There were no significant differences in survival rate among the group (78.42% ± 1.29%).

The seawater used in the experiment was filtered using a composite sand filter. The chamfer was shaded to preserve temperature and control light. The culture conditions were maintained at 29℃ ± 1℃, pH 7.5–8.0, dissolved oxygen >5.0 mg/L, salinity 29 –31. Biological preparations, such as photosynthetic bacteria, were added to the aquaria ponds every seven days (Li et al., 2016).

The special formula feed for shrimp (contain 40% crude protein, 8% crude lipid) was purchased from a local commercial feed company. This feed was mainly made of fish meal, fermented soybean meal, vitamin, etc. All shrimp groups were fed daily at a rate of 6%‐10% of body weight, divided into two equal (at time 8:30 and 16:30). The daily feeding dose was recalculated and adjusted every 15 d. The feeding experiment lasted for 90 days. Diets were hand‐fed until apparent satiation. The feeding rates were selected to assure apparent satiation was reached without overfeeding (Zhou et al., 2013). Sewage was discharged from the central sump 2 hr after feeding, followed by the addition of 10% fresh seawater.

### 2.2 Sample collection

After the 90‐day feeding trial, shrimps in each pond were sampled 24 hr after the last feeding. 30 shrimps from each pond (20.10 ± 2.30 g) were randomly selected to analyze approximate meat composition. All samples were kept on ice until they reached the laboratory. The abdominal meat was obtained from the shell, got rid of the intestinal tract, pooled in triplicate, and homogenized using a grinder. The samples were stored in PE bags in the dark at 20℃ until analysis (within 2 w).

All chemicals and solvents used were analytical grade (Sinopharm, China). Fatty acid methyl esters (FAME, purity≥98%) and mixed standards of amino acids (purity≥99%) were purchased from Sigma‐Aldrich.

### 2.3 The determination of proximate composition

Crude protein, crude lipid, moisture, and ash content in the shrimp meat were determined following the procedures of the Association of Official Analytical Chemists (AOAC, 1995). First, the samples were oven‐dried until a constant weight was reached at 105℃ to determine the moisture content. Crude protein content was estimated using the Kjeldahl method (Nitrogen Analyzer 8,400, Foss, Denmark), and a conversion factor of 6.25 was used to convert total nitrogen to crude protein. Crude lipid was assayed by ether extraction method using the Soxhlet extraction method (Sotex‐8000, Foss, Denmark). The ash content was determined by dry ashing in a muffle oven at 550℃ for 24 hr.

### 2.4 The determination of hydrolyzed amino acid (HAA) and free amino acid (FAA)

HAA content was measured according to the GB/T 5,009.124–2016 in China (2016). The sample was hydrolyzed with 6 mol L mol/L HCl at 110℃ for 24 hr in sealed glass tubes filled with nitrogen; then, 1 ml of hydrolysate was evaporated to dryness at 45℃ to remove the HCl. The hydrolysate was dissolved in 5 ml of 0.02 mol/L HCl, centrifuged (5,000 rpm, 10 min, 4℃), and then filtered. 1 µl aliquot of the supernatant was used for the amino acid analysis. Cysteine and methionine were determined as cysteic acid and methionine sulfone, respectively, by performic acid oxidation prior to their digestion in 6 N HCl. The content of tryptophan was determined after alkaline hydrolysis (5 mol/L NaOH) of each sample. All determinations were performed in triplicate.

Free amino acid (FAA) was carried out using the method of Li et al., (2014) with some modification. The sample was mixed with 0.01 mol/L HCl, supersonic extracted for 30 min, and then filtered. 1 µl aliquot of the supernatant was used for the amino acid analysis.

The amino acid composition was analyzed using an automatic amino acid analyzer (LA‐8080, Hitachi, Tokyo, Japan) equipped with a 4.6 mm ×60 mm Hitachi 2,622 column. The identity and quantity of the amino acids were assessed by comparison with the retention times and peak areas of amino acid standards. Each sample was measured thrice and presented as the average of three replicates.

The essential amino acid score was calculated with respect to the Food and Agriculture Organization/World Health Organization reference amino acid pattern of the preschool child (FAO/WHO/UNU, 1985) using the following equation:

Amino acid score =sample amino acid/reference amino acid ×100.

### 2.5 The determination of fatty acid

Fatty acids were extracted from the shrimp samples and analyzed according to the ISO 5509 method (2000) involving Soxhlet extraction, saponification, esterification, and finally fatty acid methyl ester (FAME) extraction in hexane. Gas chromatography was performed using an Agilent Technologies 6,890 gas chromatograph equipped with an HP‐5 cross‐linked methyl silicone‐fused‐silica capillary column (30 m × 0.32 mm I.D., 0.5 µm film thickness). Individual components were identified by comparing the mass spectral data and retention time data with those obtained for authentic and laboratory standards. Each sample value represents the mean of three measurements.

### 2.6 Statistical analysis

All results were expressed as mean ± *SD*, and one‐way analysis of variance (ANOVA) was performed using statistical analysis software (SPSS Version 16.0). Differences in the concentration of nutritional elements among shrimp strains were tested with ANOVA followed by a multiple comparison test (Tukey's HSD). Differences were considered to be significant at *p* <.05.

### 2.7 Ethical statement

The Pacific white shrimp is an aquaculture species. All experiments in this study were approved by the Animal Care and Use guideline of the Marine Science Research Institute of Shandong Province.

## 3 RESULTS AND DISCUSSION

### 3.1 Proximate composition of four shrimp strains

The proximate composition of each strain is shown in Table 1. Crude protein content ranged from 21.1% to 22.3%, crude fat content ranged from 0.8% to 1.1%, and the crude ash did not differ among strains. These results are consistent with Ma et al. (2018) who found that crude protein and fat content were relatively uniform among shrimp strains. Among the four strains examined in the present study, crude protein content was the highest in Universal (22.3%). Crude fat content was the lowest in common shrimp, and crude ash content was the lowest in KH‐1 and common shrimp. The samples came from the same culture area, but results were found to be comparable. A similar crude protein content was found in *Aristaeomorpha foliacea* (Bonoet al., 2012) and *Penaeus monodon* (Sriket et al., 2007) (87.7 g 100g^‐^^1^ dry matter), while *Fenneropenaeus chinensis* had a low crude protein content of 20.60% (Wang et al., 2013). Shrimp cultured in pond had higher values of crude fat, as reported by Sriket et al. (2007). In general, the chemical composition analyses confirmed that the four strains were to be excellent food resources, with good balance of nutrients and good level of protein.

**TABLE 1:** Proximate composition of four shrimp strains (mean±*SD*) (g 100g^‐1^ wet weight)

Strains	Moisture	Protein	Fat	Ash	Energy
Universal	74.6 ± 0.51b	22.3 ± 0.30a	1.1 ± 0.02a	1.7 ± 0.02a	425 ± 1.2a
KH−1	75.4 ± 0.39b	21.8 ± 0.31ab	1.0 ± 0.01b	1.6 ± 0.03a	411 ± 0.9b
Syaqua	75.2 ± 0.43ab	21.7 ± 0.29ab	1.0 ± 0.02b	1.7 ± 0.02a	413 ± 1.5b
Common	76.5 ± 0.41a	21.1 ± 0.34b	0.8 ± 0.01c	1.6 ± 0.03a	388 ± 1.4c

Notes

Values in the same column bearing different letters are significantly different (*p* <.05).

### 3.2 HAA profiles of four shrimp strains

The hydrolyzed amino acid profiles (HAA) of four shrimp strains are presented in Table 2. In all strains, glutamic acid was the most abundant amino acid, followed by lysine, aspartic acid, and leucine. These four amino acids constituted 41.34%‐42.64% of the total amino acids (TAA). The KH‐1 had the highest TAA amounts at 82.07 g 100g^‐^^1^ (DW). Additionally, the essential amino acid (EAA) was high. The ratio of EAA/TAA ranged from 38.22% (common) to 38.79% (Syaqua). The ratio of DAA/TAA ranged from 8.13% (Universal) to 8.90% (common). The ratio of EAA/NEAA ranged from 61.52% (KH‐1) to 63.38% (Syaqua).

**TABLE 2:** Amino acid composition (hydrolyzed and free) of four shrimp strains (mean±*SD*) (g 100g^‐1^ dry weight)

Amino acid	Hydrolyzed amino acid				Free amino acid			
	Universal	KH−1	Syaqua	Common	Universal	KH−1	Syaqua	Common
Aspartic acid ^#^	6.16 ± 0.01b	6.50 ± 0.01c	6.73 ± 0.02b	6.90 ± 0.01a	0.201 ± 0.001b	0.203 ± 0.001a	0.161 ± 0.001b	0.128 ± 0.000c
Threonine ^*^	3.14 ± 0.01a	3.21 ± 0.01a	3.27 ± 0.03a	3.06 ± 0.02a	0.406 ± 0.002a	0.447 ± 0.009a	0.403 ± 0.009a	0.170 ± 0.001a
Serine	2.71 ± 0.02ab	2.80 ± 0.02b	2.74 ± 0.02ab	2.86 ± 0.01a	0.051 ± 0.00b	0.122 ± 0.002a	0.040 ± 0.001b	0.043 ± 0.001b
Glutamine ^#^	13.98 ± 0.01ab	14.19 ± 0.02b	14.40 ± 0.05a	13.88 ± 0.01ab	2.028 ± 0.010b	1.748 ± 0.009a	1.573 ± 0.010b	0.468 ± 0.009c
Glycine ^#^	5.47 ± 0.02c	6.38 ± 0.01b	5.65 ± 0.01c	7.10 ± 0.01a	1.976 ± 0.018c	1.951 ± 0.015b	1.855 ± 0.012b	2.638 ± 0.014a
Alanine ^#^	5.95 ± 0.01c	6.34 ± 0.01a	6.21 ± 0.02b	5.45 ± 0.01d	1.874 ± 0.025b	1.910 ± 0.031a	1.694 ± 0.027ab	0.851 ± 0.019c
Cysteine	4.42 ± 0.01a	1.38 ± 0.02a	1.37 ± 0.03a	1.33 ± 0.01a	0.201 ± 0.003a	0.122 ± 0.004b	0.121 ± 0.001b	0.128 ± 0.001b
Valine	3.78 ± 0.02a	3.86 ± 0.01a	3.91 ± 0.01a	3.49 ± 0.02b	0.457 ± 0.005b	0.488 ± 0.009a	0.444 ± 0.009a	0.170 ± 0.005c
Methionine ^*^	2.76 ± 0.01b	2.76 ± 0.01b	2.74 ± 0.01b	3.37 ± 0.01a	0.201 ± 0.001c	0.244 ± 0.001a	0.202 ± 0.002b	0.170 ± 0.001c
Isoleucine ^*^	3.62 ± 0.02a	3.82 ± 0.02a	3.75 ± 0.01a	3.33 ± 0.03b	0.303 ± 0.001a	0.325 ± 0.001a	0.282 ± 0.001a	0.085 ± 0.001a
Leucine ^*^	6.50 ± 0.01a	6.54 ± 0.03a	6.69 ± 0.01a	6.08 ± 0.02b	0.559 ± 0.003c	0.650 ± 0.008a	0.565 ± 0.010b	0.170 ± 0.002d
Tyrosine	2.60 ± 0.04a	2.52 ± 0.01a	2.66 ± 0.02a	2.67 ± 0.01a	0.051 ± 0.001b	0.081 ± 0.001a	0.041 ± 0.001b	0.043 ± 0.001b
Phenylalanine ^*^	3.46 ± 0.01a	3.54 ± 0.01a	3.59 ± 0.03a	3.49 ± 0.02a	0.354 ± 0.002c	0.447 ± 0.003a	0.363 ± 0.004b	0.128 ± 0.001d
Lysine ^*^	6.89 ± 0.02ab	6.71 ± 0.02c	6.94 ± 0.05bc	7.10 ± 0.01a	0.606 ± 0.005b	0.691 ± 0.006a	0.524 ± 0.007b	0.298 ± 0.002c
Histidine^△^	1.73 ± 0.02a	1.75 ± 0.01a	1.77 ± 0.02a	1.73 ± 0.01a	0.201 ± 0.001b	0.203 ± 0.001a	0.161 ± 0.001b	0.085 ± 0.001c
Tryptophan^*^	0.83 ± 0.01a	0.81 ± 0.01a	073 ± 0.01a	0.74 ± 0.02a	0.039 ± 0.00a	0.041 ± 0.001a	0.040 ± 0.001a	0.043 ± 0.00a
Arginine^△^	5.24 ± 0.01a	4.80 ± 0.03c	4.96 ± 0.01b	5.22 ± 0.01a	0.205 ± 0.001a	0.122 ± 0.002b	0.121 ± 0.001b	0.043 ± 0.000c
Proline	3.39 ± 0.02b	4.14 ± 0.01a	3.39 ± 0.02b	2.43 ± 0.03c	0.472 ± 0.010b	1.138 ± 0.012a	0.766 ± 0.009c	0.043 ± 0.001d
Total amino acids (TAA)	80.08 ± 0.06ab	82.07 ± 0.05c	81.49 ± 0.06bc	80.24 ± 0.05a	7.992 ± 0.260c	10.894 ± 0.180a	9.315 ± 0.200ab	5.600 ± 0.280d
Essential amino acids (EAA)	30.98 ± 0.03a	31.26 ± 0.02a	31.61 ± 0.02a	30.67 ± 0.02a				
Delicious amino acids (DAA)	32.01 ± 0.02c	33.42 ± 0.05b	32.98 ± 0.03bc	33.33 ± 0.02a				
EAA/TAA	38.69%	38.09%	38.79%	38.22%				
EAA/NEAA	63.11%	61.52%	63.38%	61.86%				
DAA/TAA	39.97%	40.71%	40.48%	41.54%				

Notes

indicates delicious amino acids; ^*^ indicates essential amino acids.^△^ indicates semi‐essential amino acids. Values in the same column bearing different letters are significantly different (*p* <.05).

The nutritional value of food depends on amino acid composition and content. For humans, food with higher amino acid content can provide better nutritional value (Shi et al., 2013). TAA and EAA are also important indicators of the nutritional value of aquatic products (Wu et al., 2014). The four shrimp strains evaluated in this study had glutamic acid, content ranging from 172.8 mg g^‐^^1^ (KH‐1) to 176.6 mg g^‐^^1^ (Syaqua) crude protein. These values are significantly higher than those reported for fish: 114 mg/g crude protein in *Pampus punctatissimus* (Zhao et al., 2010), 117 mg/g crude protein in *Paralichthys olivaceus* (Ning et al., 2018); crabs: 147 mg/g crude protein in *Carcinus maenas* (Naczk et al., 2004), 151 mg/g crude protein in *Eriocheir sinensis* (Chen et al., 2007); and other shrimps: 135 mg/g crude protein in *Euphausia superba* (Li et al., 2014). Glutamate contributes to biochemical metabolism in brain tissue, synthesizing active substances and playing an important role in synaptic transmissions between nervous systems (Morris et al., 1987). Furthermore, the efflux of glutamic acid from meat in response to critical illness can serve as an important carrier of ammonia (nitrogen) to the splanchnic area and the immune system (Deutz et al., 1992). The shrimp protein was also rich in lysine, which is the limiting amino acid in the cereal‐based diets of children in developing countries (Kim et al., 2000).

The EAA scores of each shrimp strain are presented in Table 3. When compared to the reference amino acid pattern of preschool children (2–5 years old), all amino acid scores were >100, except for tryptophan. Tryptophan had the lowest scores (especially in Syaqua), which would be considered the limiting amino acid in shrimp. However, in Chinese recipes, shrimp is often cooked with pork, mutton, and millet, which will supply the limiting amino acid (Jilin and Peck, 1995). Lysine and sulfur‐containing amino acids (cysteine and methionine) had the highest scores in all strains. In general, the shrimp muscle protein was of high quality and exhibited well‐balanced EAA compositions.

**TABLE 3:** Essential amino acids scores in four shrimp strains

Amino acid	Reference*	Universal		KH−1		Syaqua		Common	
		content	score	content	score	content	score	content	score
Threonine	34	39.33	115.68	39.13	115.09	40.08	117.88	38.12	112.12
Tryptophan	11	10.32	93.82	9.91	90.09	8.91	81.00	9.29	84.45
Cysteine+Methionine	25	52.11	208.44	50.52	202.08	50.47	201.88	58.65	234.60
Valine	35	47.20	134.86	47.05	134.43	47.99	137.11	43.50	124.29
Phenylalanine+Tyrosine	63	75.70	120.16	73.80	117.14	76.70	121.74	76.74	121.81
Isoleucine	28	45.23	161.54	46.56	166.29	46.02	164.35	41.54	148.36
Leucine	66	81.12	122.91	79.74	120.82	82.14	124.45	75.76	114.79
Lysine	38	86.04	226.42	81.72	215.05	85.10	223.95	88.47	232.82
Average score			147.97		145.12		146.55		146.65

* Reference amino acid pattern of preschool children (2–5 years) (FAO/WHO/UNU, 1985) (mg/g protein).

### 3.3 FAA profiles of four shrimp strains

The free amino acid (FAA) profiles of the four shrimp strains are presented in Table 2. The total FAA contents were 7.99 g 100g^‐1^ (DW), 10.89 g 100g^‐1^ (DW), 9.32 g 100g^‐1^ (DW), and 5.60 g 100g^‐1^ (DW), respectively. The most abundant FAA was glutamic acid, glycine, alanine, and lysine, which differed from the HAA profiles. These amino acids constituted 63.05%, 57.83%, 60.61%, and 75.10% of the total FAA (TFAA), respectively. The least abundant FAA in the meat of all four shrimp strains was tryptophan.

The taste activity value (TAV) of the FAA identified in the four shrimp strains is presented in Table 4. FAAs are important components of the meat that can affect the flavor of aquatic animals (Li et al., 2014). Each amino acid has a unique taste, the flavor contribution of which is dependent on the TAV, the ratio between the taste substance contained in the sample and its threshold value (Chen et al., 2012; Li et al., 2014). A TAV >1 indicates an important contribution to the taste of food (Wang and Chen, 2014). However, TAV does not consider the interaction between the components, which may have a synergistic effect on food taste. Maybe sensory evaluation should be used in our future research. Among the four shrimp strains, glutamic acid, glycine, valine, methionine, lysine, and histidine had TAVs greater than one. Amino acids, especially glutamic acid, glycine, alanine, and aspartic acid greatly influence the shrimp flavor (Liu et al.,l., 2017).

**TABLE 4:** The taste attribute and TAV of free amino acid in four shrimp strains (mg g^‐1^)

Amino acid	Taste attribute	Threshold value	TAV			
			Universal	KH−1	Syaqua	Common
Aspartic acid	sweet/sour (+)	1	0.40	0.50	0.40	0.30
Threonine	sweet (+)	2.6	0.31	0.42	0.38	0.15
Serine	sweet (+)	1.5	0.07	0.20	0.07	0.07
Glutamine	sweet/sour (+)	0.3	13.33	14.33	13.00	3.67
Glycine	sweet (+)	1.3	3.00	3.69	3.54	4.77
Alanine	sweet (+)	0.6	6.17	7.83	7.00	3.33
Valine	sweet/bitter (‐)	0.4	2.25	3.00	2.75	1.00
Methionine	bitter/sweet/sulfurous (‐)	0.3	1.33	2.00	1.67	1.33
Isoleucine	bitter (‐)	0.9	0.67	0.89	0.78	0.22
Leucine	bitter (‐)	1.9	0.58	0.84	0.74	0.21
Phenylalanine	bitter (‐)	0.9	0.78	1.22	1.00	0.33
Lysine	sweet/bitter (‐)	0.5	2.40	3.40	2.60	1.4
Histidine	bitter (‐)	0.2	2.00	2.50	2.00	1.00
Arginine	bitter/sweet (‐)	0.5	0.80	0.6	0.60	0.20
Proline	bitter/sweet (‐)	3	0.4	0.93	0.63	0.03

**FIGURE 1:**
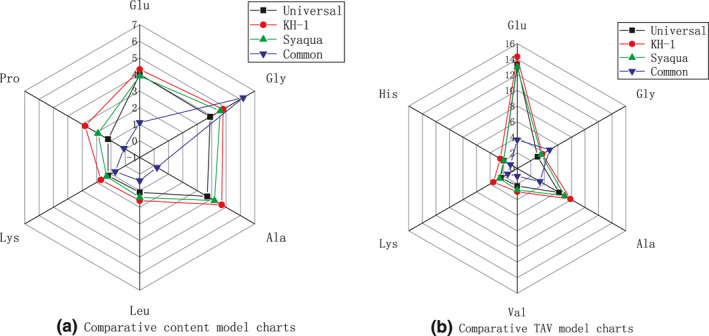
Comparative content and comparative TAV model charts of major free amino acids Abbreviations: Glu, glutamine; Gly, glycine; Ala, alanine; Val, valine; Leu, leucine; Lys, lysine; His, histidine

Figure 1 shows the comparative content model charts and TAV model charts of the major FAA in the four shrimps, respectively. All strains had the similar FAA compositions, except the common shrimp, which had higher glycine levels (Figure 1a). The FAA content model among the other three strains was similar, but the model area was highest for KH‐1, followed by Syaqua, and then Universal, which indicates that the FAA content of KH‐1 was higher.

The FAA content models were similar among strains, except for the common shrimp (Figure 1b). KH‐1 had the largest model area. The top contributor to taste was glutamine, with a TAV of 14.33. The TAVs of alanine and glycine were 7.83 and 3.69, respectively, which also significantly influence the taste of shrimp meat. The models indicate that KH‐1 would taste fresher and sweeter than the other strains.

### 3.4 Fatty acid profiles of four shrimp strains

The fatty acid profiles and levels, which varied among the four strains, are shown in Table 5. The profiles of all strains were dominated by polyunsaturated fatty acids (PUFA) except in the common shrimp, followed by saturated fatty acids (SFA), and monounsaturated fatty acids (MUFA). Among the SFAs, palmitic acid (C16:0) was the dominant and stearic acids (C18:0) were also abundant. The dominant MUFA was oleic acid (C18:1). The dominant PUFAs were linoleic acid (LA, C18:2–6), docosahexaenoic acid (DHA, C22:6–3), and eicosapentaenoic acid (EPA, C20:5–3).

**TABLE 5:** Fatty acid profile (g/100g wet weight) of four shrimp strains (mean±*SD*) (g 100g^‐1^)

Fat acid	Universal	KH−1	Syaqua	Common
C4:0	0.04 ± 0.00a	0.02 ± 0.00a	0.03 ± 0.00a	0.04 ± 0.00a
C6:0	0.02 ± 0.00a	‐	0.02 ± 0.00a	0.03 ± 0.00a
C10:0	0.05 ± 0.00a	0.04 ± 0.00a	0.06 ± 0.00a	0.29 ± 0.00a
C11:0	7.29 ± 0.18c	7.03 ± 0.05c	9.47 ± 0.09b	10.98 ± 0.05a
C12:0	0.06 ± 0.00b	0.04 ± 0.00c	0.06 ± 0.00b	0.08 ± 0.01a
C14:0	0.61 ± 0.01a	0.34 ± 0.00c	0.31 ± 0.00d	0.45 ± 0.01b
C15:0	0.77 ± 0.00b	1.08 ± 0.09a	0.66 ± 0.01bc	0.63 ± 0.00c
C16:0	16.83 ± 0.10b	16.75 ± 0.07b	15.94 ± 0.09c	17.46 ± 0.10a
C17:0	1.41 ± 0.01d	1.99 ± 0.02b	1.62 ± 0.01c	2.64 ± 0.05a
C18:0	10.23 ± 0.19a	10.32 ± 0.20a	10.08 ± 0.21a	9.05 ± 0.15b
C20:0	0.72 ± 0.01a	0.67 ± 0.01b	0.57 ± 0.02c	0.65 ± 0.01b
C21:0	0.22 ± 0.00	0.20 ± 0.00	0.15 ± 0.00	0.22 ± 0.00
C22:0	0.98 ± 0.01a	0.76 ± 0.00b	0.70 ± 0.01c	0.98 ± 0.00a
C23:0	0.20 ± 0.00b	0.21 ± 0.00b	0.18 ± 0.00c	0.29 ± 0.01a
C24:0	0.29 ± 0.00	0.28 ± 0.00	0.21 ± 0.00	0.36 ± 0.00
∑SFA	39.72 ± 0.26b	39.73 ± 0.31b	40.06 ± 0.41b	44.15 ± 0.38a
C16:1(*n*−7)	2.10 ± 0.02b	1.37 ± 0.01c	1.01 ± 0.00d	3.77 ± 0.12a
C18:1(trans,*n*−9)	0.56 ± 0.00	0.41 ± 0.00	‐	0.61 ± 0.00
C18:1(cis,*n*−9)	13.89 ± 0.21a	13.10 ± 0.09b	12.71 ± 0.09c	12.40 ± 0.07c
C20:1	0.69 ± 0.00b	0.72 ± 0.00a	0.72 ± 0.01a	0.37 ± 0.00c
C24:1(*n*−9)	0.43 ± 0.00	0.30 ± 0.00	0.35 ± 0.00	0.33 ± 0.00
∑MUFA *n*−9	14.88 ± 0.07a	13.81 ± 0.08b	13.06 ± 0.06d	13.34 ± 0.10c
∑MUFA	17.67 ± 0.09a	15.90 ± 0.08b	14.79 ± 0.11c	17.48 ± 0.05a
C18:2(LA,*n*−6)	16.12 ± 0.07a	15.12 ± 0.07b	16.14 ± 0.08a	9.49 ± 0.11c
C20:2	1.34 ± 0.01c	1.61 ± 0.05b	1.85 ± 0.09a	0.77 ± 0.06d
C22:2	0.04 ± 0.00a	0.05 ± 0.00a	0.07 ± 0.00a	0.10 ± 0.00a
C18:3(GLA,*n*−6)	0.05 ± 0.00	0.07 ± 0.00	0.04 ± 0.00	0.23 ± 0.00
C18:3(ALA,*n*−3)	1.89 ± 0.05c	2.00 ± 0.04c	2.22 ± 0.07b	4.30 ± 0.05a
C20:3 (*n*−6)	0.07 ± 0.00	0.10 ± 0.00	0.08 ± 0.00	0.46 ± 0.00
C20:4 (ARA,*n*−4)	2.92 ± 0.04d	3.62 ± 0.05b	3.25 ± 0.04c	6.28 ± 0.09a
C20:5 (EPA,*n*−3)	9.56 ± 0.07c	10.35 ± 0.08a	10.09 ± 0.09b	9.93 ± 0.10b
C22:6(DHA,*n*−6)	10.60 ± 0.10b	11.44 ± 0.12a	11.14 ± 0.21a	6.55 ± 0.18c
∑PUFA *n*−3	11.45 ± 0.22c	12.35 ± 0.04b	12.31 ± 0.08b	14.23 ± 0.09a
∑PUFA *n*−6	26.77 ± 0.28ab	26.63 ± 0.19b	27.32 ± 0.25a	16.27 ± 0.19c
∑PUFA	42.59 ± 0.45b	44.36 ± 0.36a	44.88 ± 0.47a	38.11 ± 0.39c
∑PUFAn−3/∑PUFA *n*−6	0.43	0.46	0.45	0.87
∑PUFAn−6/∑PUFA *n*−3	2.34	2.16	2.22	1.14

Note

Values in the same row bearing different letters are significantly different (*p* <.05), whereas values without letters indicate no significant differences.

Abbreviations: MUFA, monounsaturated fatty acids; PUFA, polyunsaturated fatty acids; SFA, saturated fatty acids.

Lipids store and provide energy to organisms. PUFAs are essential for the reproduction and growth of organisms; they can also increase the flavor of food during heating, reduce blood viscosity, and enhance human immunity (Cui et al., 2018). Compared with pomfret (Zhao et al., 2010), Pacific white shrimp have higher amounts PUFA contents, especially the Syaqua strain. In addition, all four strains had higher MUFA levels than Chinese white shrimp *Fenneropenaeus chinensis* (Li et al., 2017). LA is a fat acid typically found in plants; the high LA content observed in shrimp may attribute to their omnivorous diets. In fact, a previous study reported higher LA content in the muscle of *L*. *vannamei* fed diets supplemented with two species of marine algae (Ju et al., 2009). Dietary lipids have also been shown to affect the fatty acid composition of *L*. *vannamei*; whole body shrimps showed increases in MUFA and PUFA when fed diets with high levels of unsaturated fatty acids.

Among the most valuable FAs, DHA and EPA play important roles in the prevention of inflammatory and cardiovascular diseases due to their serum triglycerides‐lowering effects (Bono et al., 2012). The EPA and DHA contents were moderately high in all the shrimp samples, especially KH‐1. However, the EPA content measured in the present research was lower than that reported by Huang et al., (2004) for *L. vannamei* (11.5 to 13.7 g100 g^−1^FA), while the DHA content, which ranged from 8.9 to 10.4 g 100 g^−1^ FA, was lower than that of our data.

The ratio of n‐6/n‐3 PUFAs found among the four strains in this study was lower than the threshold value recommended by the UK Department of Health (HMSO, 1994). Values>4.0 are considered to be harmful to health and may promote the development of cardiovascular disease (Moreira et al., 2001). Therefore, consuming these shrimp strains may contribute to maintenance of n‐6/n‐3 ratio recommended. The FA compositions among aquatic organisms are dependent on diet, size, age, reproductive conditions, and environmental conditions, especially water temperature, which can influence lipid content and FA composition (Zhao et al., 2010). Our results indicate that the shrimp strain influences FA content and composition.

## 4 CONCLUSION

The results from this study revealed that strain‐specific variability exists in chemical composition of the shrimp *L. vannamei* under controlled condition. The Universal strain had the highest protein and fat content, and the highest overall EAA score. The Syaqua shrimps showed the highest levels of PUFA. And the KH‐1 strain was highest in FAA taste‐active components.

## CONFLICTS OF INTEREST

The authors declare that they have no conflict of interest.

## Data Availability

The authors confirm that the data supporting the findings of this study are available within the article.
